# Molecular Characterization of a Novel Lytic Enzyme LysC from *Clostridium intestinale* URNW and Its Antibacterial Activity Mediated by Positively Charged *N*-Terminal Extension

**DOI:** 10.3390/ijms21144894

**Published:** 2020-07-11

**Authors:** Magdalena Plotka, Monika Szadkowska, Maria Håkansson, Rebeka Kovačič, Salam Al-Karadaghi, Björn Walse, Olesia Werbowy, Anna-Karina Kaczorowska, Tadeusz Kaczorowski

**Affiliations:** 1 Laboratory of Extremophiles Biology, Department of Microbiology, Faculty of Biology, University of Gdansk, 80-822 Gdansk, Poland; monika.szadkowska@phdstud.ug.edu.pl (M.S.); olesia.werbowy@ug.edu.pl (O.W.); 2 SARomics Biostructures, SE-223 81 Lund, Sweden; maria.hakansson@saromics.com (M.H.); rebkova@gmail.com (R.K.); salam.al-karadaghi@saromics.com (S.A.-K.); bjorn.walse@saromics.com (B.W.); 3 Collection of Plasmids and Microorganisms, Faculty of Biology, University of Gdansk, 80-308 Gdansk, Poland; anna.kaczorowska@ug.edu.pl

**Keywords:** bacteriophage, endolysin, autolysin, antimicrobial peptide, peptidoglycan, *Staphylococcus aureus*, thermophiles

## Abstract

Peptidoglycan hydrolytic enzymes are considered to be a promising alternative to conventional antibiotics in combating bacterial infections. To identify novel hydrolytic enzymes, we performed a database search with the sequences of two thermostable endolysins with high bactericidal activity, studied earlier in our laboratory. Both these enzymes originate from *Thermus scotoductus* bacteriophages MAT2119 and vB_Tsc2631. A lytic enzyme LysC from *Clostridium intestinale* URNW was found to have the highest amino acid sequence similarity to the bacteriophage proteins and was chosen for further analysis. The recombinant enzyme showed strong activity against its host bacteria *C. intestinale*, as well as against *C. sporogenes*, *Bacillus cereus*, *Micrococcus luteus,* and *Staphylococcus aureus*, on average causing a 5.12 ± 0.14 log reduction of viable *S. aureus* ATCC 25923 cells in a bactericidal assay. Crystallographic studies of the protein showed that the catalytic site of LysC contained a zinc atom coordinated by amino acid residues His^50^, His^147^, and Cys^155^, a feature characteristic for type 2 amidases. Surprisingly, neither of these residues, nor any other of the four conserved residues in the vicinity of the active site, His^51^, Thr^52^, Tyr^76^, and Thr^153^, were essential to maintain the antibacterial activity of LysC. Therefore, our attention was attracted to the intrinsically disordered and highly positively charged *N*-terminal region of the enzyme. Potential antibacterial activity of this part of the sequence, predicted by the Antimicrobial Sequence Scanning System, AMPA, was confirmed in our experimental studies; the truncated version of LysC (LysCΔ2–23) completely lacked antibacterial activity. Moreover, a synthetic peptide, which we termed Intestinalin, with a sequence identical to the first thirty amino acids of LysC, displayed substantial anti-staphylococcal activity with IC_50_ of 6 μg/mL (1.5 μM). This peptide was shown to have α-helical conformation in solution in the presence of detergents which is a common feature of amphipathic α-helical antimicrobial peptides.

## 1. Introduction

The discovery and introduction of antibiotics, starting with penicillin, made a revolutionary change in the way of treating bacterial infections. However, despite unquestionable benefits associated with new therapies, it was immediately observed that microorganisms quickly start to develop resistance to antibiotics and bacterial infections again become a serious threat to human life. Therefore, as the antibiotic pipeline was beginning to dry up, an urgent need emerged for novel antimicrobial agents, capable of combatting multidrug-resistant pathogens. Among the most promising alternatives to conventional antibiotics are peptidoglycan hydrolytic enzymes. Peptidoglycan (PG) is a major constituent of the bacterial cell wall with a function to protect cells from bursting due to high internal turgor pressure that can reach up to 30 atm in Gram-positive bacteria [1]. PG is built up by linear glycan strands, made up of *N*-acetylglucosaminyl-*N*-acetylmuraminyl disaccharide repeats, cross-linked by short peptide stems that together form a mesh-like structure around the bacterial cytoplasmic membrane [2]. Bacterial peptidoglycan hydrolases can be classified into three groups depending on which covalent bonds they specifically cleave in the polymer or in its soluble fragments. Amidases cleave the amide bond connecting the stem-peptide to the glycan backbone of PG, glycosidases (*N*-acetylglucosaminidases and *N*-acetylmuramidases) catalyze the hydrolysis of the glycosidic linkages, whereas peptidases cleave amide bonds between amino acids in the PG chain [3]. Based on their origin and role, peptidoglycan hydrolases can be categorized into endolysins, exolysins, and autolysins [4]. Endolysins are produced near the end of the lytic cycle of bacteriophages. They enzymatically degrade peptidoglycan ‘from within’, leading to lysis of the bacterial cell and release of phage progeny [5]. Exolysins are produced and secreted by certain bacteria to fight alien bacterial species. Examples include lysostaphin, a bacteriocin secreted by *Staphylococcus simulans* that targets *S. aureus*, including methicillin-resistant strains. Representatives of the third group, autolysins, cleave the peptidoglycan of bacteria that produce them. They are mainly involved in cellular processes, including cell growth and division, cell-wall turnover, and peptidoglycan maturation.

The functional classification of endolysins, exolysins, and autolysins, and their designation as amidases, glycosidases or peptidases, depends on the structural organization of an individual protein. Amidases, also known as *N*-acetylmuramoyl-l-alanine amidases, have three different types of catalytic domains: (i) Amidase_2 (family: PF01510), (ii) Amidase_3 (family: PF01520), and (iii) Amidase_5 (family: PF05382) [6]. The family of type 2 amidases includes zinc amidases, a representative of which is bacteriophage T7 lysozyme. This enzyme contains two conserved histidine residues, His^17^ and His^122^, and one cysteine Cys^130^ which coordinate a zinc atom [7]. Recently, our group discovered two thermostable endolysins derived from *T. scotoductus* bacteriophages MAT2119 and vB_Tsc2631. Both, Ph2119 and Ts2631 endolysins cleaved PG not only of thermophiles but also of mesophilic Gram-negative bacteria including *Escherichia coli*, *Salmonella* serovar *Panama*, *Serratia marcescens,* and *Pseudomonas fluorescens* [8,9]. However, all of the above mesophilic bacteria, before the addition of the endolysin, were treated with chloroform-saturated Tris-HCl buffer in order to permeabilize their outer membranes, which serve as a natural barrier that prevents endolysin from reaching and digesting the PG layer [10]. The difficulties associated with bypassing the outer membrane of Gram-negative bacteria for many years hindered the extracellular use of endolysins as antibacterial agents. Presently, human-safe, outer membrane permeabilizers such as a cation chelator ethylenediaminetetraacetic acid (EDTA) or weak organic acids, such as citric acid and malic acid, are used to enhance endolysin activity against Gram-negative bacteria [11]. An alternative approach to the use of permeabilizing agents is the fusion of a lytic enzyme of interest with a short peptide with membrane-penetrating capabilities.

Using molecular engineering techniques, it has already been possible to design several of such chimeric lytic enzymes. This was achieved by the addition of highly cationic nonapeptide (PCNP; a mixture of arginine and lysine residues) to OBPgp279 endolysin, which provided it with high antibacterial activity against *P. aeruginosa* [12]. The enhancement of bacteriolytic activity was also possible in the case of endolysins of Gram-positive origin. A chimeric endolysin Art-240, encompassing a fusion of the peptide PCNP with the *C*-terminus of endolysin from *Streptococcus agalactiae* prophage λ Sa2, showed an increased bactericidal activity against several streptococcal species in relation to the parental enzyme [13].

There are also examples of endolysins with natural antibacterial activity. These include recently discovered Ts2631 endolysin from thermophilic phage isolated from extreme habitat, which shows antibacterial activity against the Gram-negative pathogens, *Acinetobacter baumannii* and *Pseudomonas aeruginosa* [14]. In this case, the outer membrane permeabilization was mediated by the *N*-terminal extension of the protein that contains seven basic residues (six arginine and one lysine residues) within the first 20 amino acids.

In this study, we performed a sequence similarity search of the GenBank database to identify lytic enzymes similar to the thermostable Ph2119 and Ts2631 endolysins. From several protein candidates with a predicted lytic function, protein LysC from *C. intestinale* URNW was selected for further analysis. Here, we confirm its lytic activity against several Gram-positive bacterial species including *Staphylococcus aureus*. Upon LysC treatment, a typical lysis of staphylococcal cells, accompanied with cytoplasmic content leakage was observed under an electron microscope, together with abnormal septum formation and extensive accumulation of membranes in many cells. Crystallographic and site-directed mutagenesis studies reveal that the highly positively charged *N*-terminal region of the protein is the major contributor to the antimicrobial activity of LysC. This finding is also supported by bioinformatics analysis and other experimental data. A 30 amino acids peptide, which we termed Intestinalin, designed and synthetized based on the sequence of the *N*-terminal region of LysC, was shown to have high antibacterial activity against *S. aureus* ATCC 25923. This discovery paves the way for the development of a new class of agents with superb antibacterial activity.

## 2. Results

### 2.1. Bioinformatics

**Figure 1:**
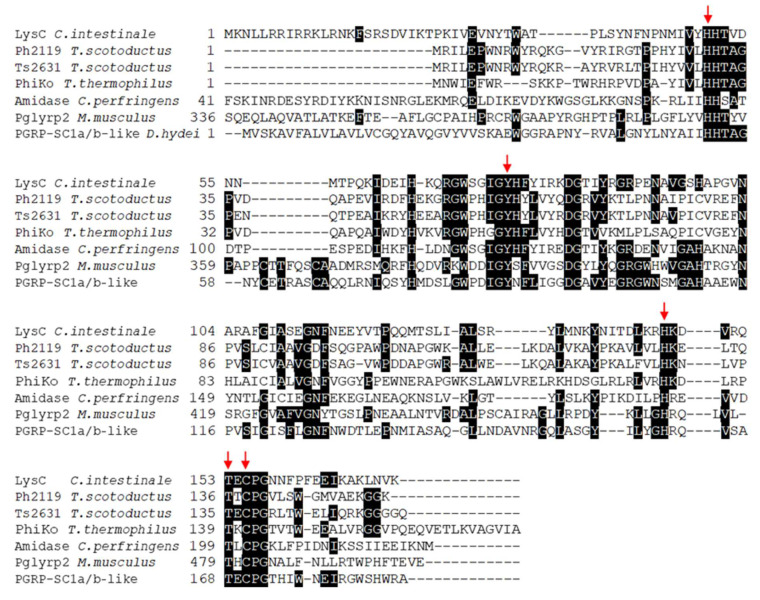
Multiple-sequence alignment of amino acid sequences of LysC lytic enzyme, Ph2119 and Ts2631 endolysins, *N*-acetylmuramoyl-l-alanine amidase from *Clostridium perfringens,* and eukaryotic peptidoglycan recognition proteins. The alignment was performed using Clustal Omega. Black boxes highlight conserved amino acids. LysC from *C. intestinale* URNW with conserved residue pattern similar to that of the thermostable Ph2119 endolysin was chosen for further analysis. The red arrows indicate conserved residues His50, Tyr76, His147, Thr153, and Cys155 of LysC, which are indispensable for amidase activity (https://www.ncbi.nlm.nih.gov/protein/ERK30183.1).

The basic local alignment search tool (BLASTP) sequence-similarity searches for proteins showing homology to eukaryotic peptidoglycan recognition proteins (PGRPs) and thermostable Ph2119 and Ts2631 endolysins allowed to identify in the genome of *Clostridium intestinale* URNW (accession no APJA01000012.1) an open reading frame (ORF, coordinates 584335-584853 nt) encoding a 172 aa protein (M_r_ = 20,090; pI = 9.96) with a putative lytic function. The enzyme (accession no ERK30183.1) shows 34% amino acid sequence identity (E value 5e-21) to the thermostable Ph2119 endolysin from *Thermus scotoductus* bacteriophage MAT2119 and 33% identity (E value 6e-19) to the Ts2631 endolysin from *T. scotoductus* bacteriophage vB_Tsc2631. Both lytic enzymes were analyzed in detail in our laboratory [8,9,14,15]. The LysC protein also resembles a putative lysozyme from *Thermus thermophilus* bacteriophage PhiKo (AYJ74695.1), several *N*-acetylmuramoyl-l-alanine amidases (EC: 3.5.1.28) from various *Clostridium* species including *C. perfringens* (PWX13837.1) and eukaryotic recognition proteins, PGRPs. BLASTP analysis indicates that LysC contains a conserved *N*-acetylmuramoyl-l-alanine amidase domain, and similarly to Ph2119 endolysin, belongs to the PGRP family (accession cd06583). However, unlike the thermostable endolysin, LysC also gives a specific hit to the AmpD family (accession COG3023), which groups *N*-acetyl-anhydromuramyl-l-alanine amidases involved in cell wall/membrane/envelope biogenesis [16,17]. The alignment of the amino acid sequences of the LysC, thermostable endolysins, *N*-acetylmuramoyl-l-alanine amidase from *C. perfringens,* and representatives of eukaryotic PGRPs, is shown in Figure 1. In the figure, red arrows highlight the invariant residues of the characteristic endolysin motifs—the HHTAG motif in the *N*-terminal region, IGYH motif in the middle, and T(X)CPG motif in the *C*-terminal part, where variable X represents glutamic acid (E), threonine (T), or lysine (K) residues in sequences of LysC/Ts2631, Ph2119, and PhiKo lysozyme, respectively. The resemblance of unexplored LysC to endolysins with high antibacterial potential makes the protein an interesting experimental model for detailed analysis.

To test whether *lysC* gene, similarly to genes coding for Ph2119 and Ts2631 endolysins, is of phage origin, the contig 00012 of the whole genome sequence (APJA01000012.1) of *C. intestinale* strain URNW was analyzed by the online tool PHAge Search Tool Enhanced Release (PHASTER) [18]. Only one intact 64.7 Kb prophage region (coordinates 461851–526605 nt) restricted by two attachment sites attL and attR was identified. Genomic localization of the *lysC* gene (coordinates 584335–584853 nt), separated by more than 68 kb from the nearest annotated phage gene, indicates that it probably encodes a bacterial autolysin involved in peptidoglycan remodeling rather than a phage lytic enzyme. The identical gene (610419–610937) is also present in *C. intestinale* DSM 6191 genome (NZ_FQXU01000003), and similarly, it is not located within any predicted prophage region (data not shown).

### 2.2. LysC Characterization and Activity Assays

**Figure 2:**
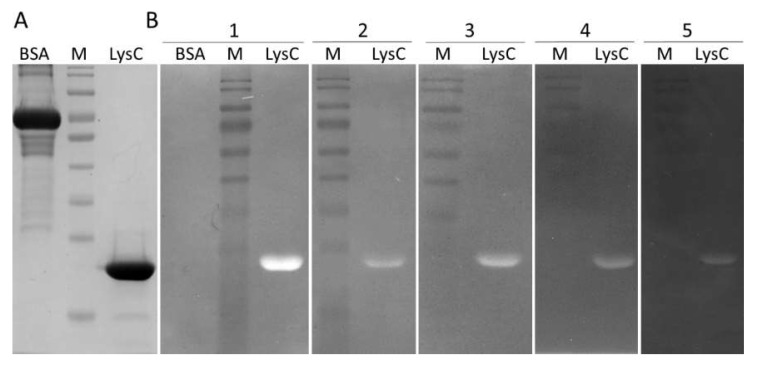
The SDS-PAGE profile and zymogram analysis of the lytic activity of LysC. **(A)** SDS-PAGE (12.5%) of 1 μg of bovine serum albumin (BSA) and purified LysC; **(B)** Zymogram analysis showing lytic activity of LysC against (**1**) *C. intestinale* DSM 6191, (**2**) *C. sporogenes* DSM 767, (**3**) *B. cereus* ATCC 13061, (**4**) *M. luteus* ATCC 4698, and (**5**) *S. aureus* ATCC 25923; BSA in panel 1 served as a negative control; Lytic activity of LysC is indicated by a single cleared band. M: PageRuler pre-stained protein ladder, 10 to 180 kDa (Thermo Fisher Scientific).

Recombinant LysC was overproduced in *E. coli* and purified to near homogeneity (Figure 2A). The oligomeric state of the protein was investigated by size-exclusion chromatography. As shown in Figure S1, the protein eluted as a single peak at a volume of 13.02 mL, which corresponds to a molecular weight of 23,380. This value is only 1130 higher than that calculated by ProtParam for the recombinant protein (M_r_ = 22,250), suggesting that LysC exists in solution in a monomeric form.

The lytic activity of the protein was determined by zymogram assay (Figure 2B). LysC was able to digest *C. intestinale* cells embedded in the zymogram gel, generating a clearing band (Figure 2B(1)). Subsequently, the zymogram assay was used to screen the activity of LysC against several Gram-positive bacteria. The lytic band was observed for *C. sporogenes* DSM 767 (Figure 2B(2)), *B. cereus* ATCC 13061 (Figure 2B(3)), *M. luteus* ATCC 4698 (Figure 2B(4)), and *S. aureus* ATCC 25923 (Figure 2B(5)). No lysis was observed for *C. perfringens* strain Cp39 (S. M. Swift, personal communication) and *B. subtilis* 168 DSM 23778 (data not shown). Bovine serum albumin (BSA, 5 μg) served as a negative control in this assay (Figure 2A) and as expected, no clearing zone was observed in the zymogram gel (Figure 2B(1)).

**Figure 3:**
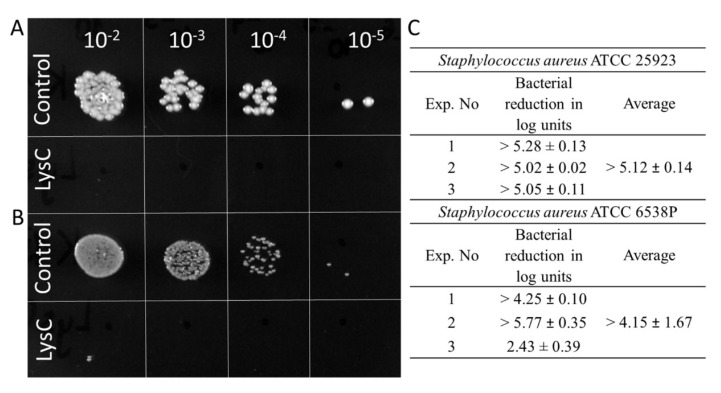
Antimicrobial activity of LysC. (**A**) Activity against *S. aureus* ATCC 25923; (**B**) Activity against *S. aureus* ATCC 6538P. Both bacteria (>10^5^) were mixed with LysC dissolved in 20 mM HEPES buffer, pH 7.4, to a final volume of 300 μL and a final enzyme concentration of 500 μg/mL. The mixtures were incubated at 37 °C for 1.5 h. Aliquots of 5 μL, containing serial 10-fold dilutions prepared in 20 mM HEPES buffer, pH 7.4, were spotted onto tryptic soy broth (TSB) agar plate. The plate was incubated overnight, and then photographed; (**C**) Serial 10-fold dilutions of controls and reactions with LysC, in a volume of 100 μL were spread onto TSB agar plates. After an overnight incubation at 37 °C, colony forming units (CFUs) were counted and the antibacterial activity was reported as bacterial reduction in logarithmic units relative to the untreated control.

Since the results of the zymograms show the lytic effect of LysC against *S. aureus*, an important human pathogen causing a range of clinical infections [19], the next step was to quantify the anti-bacterial activity of LysC by colony forming unit (CFU) reduction assay. Two strains of *S. aureus* ATCC 25923 and ATCC 6538P were chosen for this analysis (Figure 3). Spot tests showed total eradication of *S. aureus* ATCC 25923 cells and the presence of single colonies of *S. aureus* ATCC 6538P after 1.5 h incubation with 500 µg/mL of LysC at 37 °C (Figure 3A,B). On average, LysC reduced the cell number of *S. aureus* ATCC 25923 by more than 5.12 ± 0.14 log units whereas a reduction of more than 4.15 ± 1.67 in cell number was observed for *S. aureus* ATCC 6538P (Figure 3C). However, bacterial colonies of the latter strain after 24 h of agar plates incubation at 37 °C were clearly smaller than that of *S. aureus* ATCC 25923 (Figure 3B). Moreover, it was difficult to cultivate the *S. aureus* ATCC 6538P strain in a standardized way, which resulted in lower reproducibility of bactericidal assay results for this strain (Figure 3C).

### 2.3. Evaluation of LysC Activity by An Electron Microscopy

**Figure 4:**
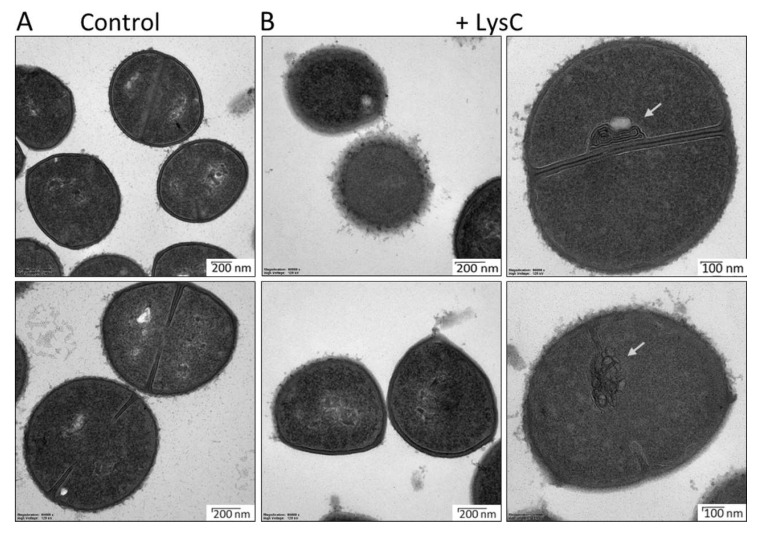
Transmission electron microscopy of *Staphylococcus aureus* ATCC 25923 cells treated with LysC enzyme. (**A**) Untreated cells of *S. aureus* ATCC 25923; (**B**) *S. aureus* ATCC 25923 treated with LysC (500 μg/mL) for 1.5 h at 37 °C. The dark and light areas correspond to high and low electron densities of the samples, respectively. Grey arrows show abnormal membranes accumulation, which was characteristic for many cells after incubation with LysC.

Transmission electron microscopy studies were performed to visualize the antibacterial activity of LysC against *S. aureus* ATCC 25923 (Figure 4). In the control samples, round, uniformly shaped bacteria with intact cell walls were clearly visible. Incomplete or completely formed septum was noticed in many of the control cells, indicating that there were in the exponential phase of growth (Figure 4A). Addition of LysC to *S. aureus* cells led to significant morphological alterations (Figure 4B). One and half hour after incubation, many cells, while maintaining a round shape, lost the cell wall smoothness, and spherical or unstructured components protruding from the cell surface were seen. In addition, no distinction between the cytoplasmic membrane and peptidoglycan could be observed. In many cases, aberrant septum was displayed with attached mesosome-like structures, suggesting abnormal cell division. Finally, in some cells the cytoplasm content leaked out through punctures in the bacterial cell wall, however, we did not observe bacterial ghosts, characteristic for Gram-positive bacteria treated with antibacterial agents [20] (Figure 4B). Unexpectedly, the TEM results showed an increase in cell wall roughness and accumulation of membrane stacks inside LysC-treated cells, but not cell rupture and bacterial shape alterations, as was seen previously in bacteria treated with Ts2631 endolysin [14].

### 2.4. Crystal Structures of Ph2119 Endolysin and LysC

**Figure 5:**
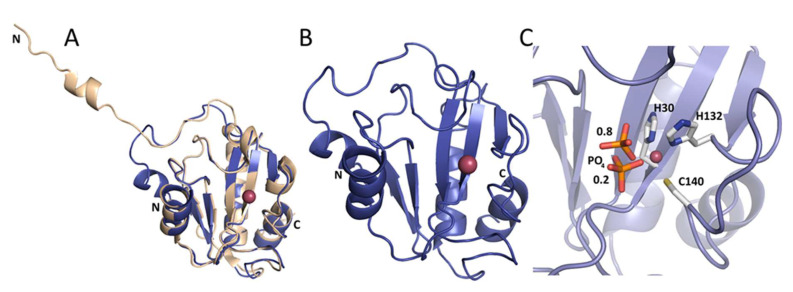
(**A**) Ts2631 endolysin (beige) superimposed on Ph2119 endolysin (blue). Unlike Ts2631, the *N*-terminus of Ph2119 folds onto itself and does not contribute to dimer stabilization. (**B**) Ph2119 endolysin with Zn^2+^ (pink sphere). (**C**) Close view of the Zn^2+^ site with coordinating side chains (H30, H132, and C140) and a phosphate group (in sticks representation) shown in two positions with occupancies of 0.8 and 0.2, as indicated by the numbers in the figure.

As a reference, we used the crystal structure of Ts2631 endolysin previously determined to 1.95 Å resolution (Protein Data Bank (PDB) entry: 6FHG). The peptidoglycan binding site of the Ts2631 protein showed an elongated cleft-like architecture with four residues coordinating a zinc atom located at the bottom of the groove—His30, Tyr58 (mediated by a water molecule), His131, and Cys139 [15]. In the present work, we determined the crystal structures of Ph2119 endolysin and LysC, both to 1.2 Å resolution. Data collection and refinement statistics are summarized in Table 1. The structure of Ph2119 endolysin was determined by molecular replacement using the coordinates of *Drosophila melanogaster* PGRP-LC as a search model (PDB entry: 2F2L). The Ph2119 endolysin, in turn, was used as a model in molecular replacement to determine the structure of LysC. The Ph2119 protein structure superimposes well on the Ts2631 endolysin structure with a root-mean-square deviation (RMSD) of 0.36 Å for 111 Cα atoms aligned (Figure 5A). In contrast to the Ts2631 enzyme, the *N*-terminus of Ph2119 endolysin does not contribute to dimer stabilization but is associated with the core domain through three hydrogen bonds (Trp7′/Lys70″, Arg9′/Glu41″, and Tyr11′/Glu48″). The Ph2119 endolysin structure displays a mixed fold of a five-stranded β-sheet (β1-β5) flanked by helices α2 and α3, one from each side, yielding a sequence of secondary structure elements α1/β1/β2/α2/β3/β4/α3/β5/α4 (Figure 5B). The active site is located in the peptidoglycan binding groove in the center of the molecule and consists of His30, His132, Cys140, and a phosphate ion (Figure 5C). The phosphate group was modeled in two adjacent positions in Ph2119 endolysin as well as in the LysC structure, with occupancies of 0.8 and 0.2. The oxygen of the phosphate group is bound to the Zn^2+^ atom, occupying the position of the peptidoglycan oxygen, when it binds during the lysis. In this way, the phosphate group may potentially block the binding site of PGs.

**Figure 6:**
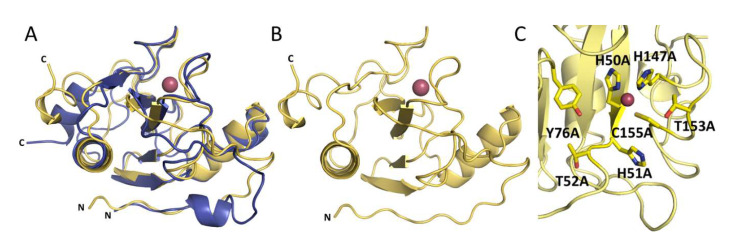
(**A**) Superposition of LysC (yellow) and Ph2119 endolysin (blue) structures. Except for the flexible *N*-terminal (*N*) and *C*-terminal (*C*) regions, the overall structures, including the Zn^2+^ sites, are conserved. (**B**) LysC with Zn^2+^ (pink sphere). (**C**) The 7 amino acids (sticks representation) around the Zn^2+^ site, which were substituted by alanine.

The overall structures of Ph2119 endolysin and LysC show conserved catalytic domain architecture, including the Zn^2+^ coordination site (Figure 6A). The overall RMSD is 0.81 Å, comparing 85 aligned Cα atoms. Unlike Ph2119 endolysin, LysC has a large part of the *N*-terminus not present in the electron density maps (23 amino acids in LysC not visible). Most probably these residues could not be traced due to high mobility-intrinsic disorder (Figure 6B). The catalytic site of LysC is composed of His50, His147, and Cys155, a motif also present in Ts2631 and Ph2119 endolysins. Other conserved residues, such as His51, Thr52 (HHT motif), Tyr76 (IGYH motif), and Thr153 (T(X)CPG motif) are close to each other in the potential peptidoglycan binding site and may therefore contribute to the catalytic function (Figure 6C). To verify this hypothesis, the seven residues shown in Figure 6C were chosen for site-directed mutagenesis studies to assess their involvement in LysC catalytic activity.

### 2.5. Antibacterial Activity of LysC Single-Residue Substitution Variants

Seven residues, His50, His51, Thr52, Tyr76, His147, Thr153, and Cys155, were substituted with alanine, yielding seven different variants of the enzyme (Figure 6C). LysC substitution variants were obtained by site-directed mutagenesis with the use of pET-LysC plasmid with primary *lysC* gene as a template and mutagenic primers that are listed in Table S1. The sequencing results showed that all recombinant plasmids were successfully constructed. Resulted LysC variants H50A, H51A, T52A, Y76A, H147A, T153A, and C155A (zinc coordinating residues are in bold) were heterologously expressed in *E. coli* BL21(DE3) and purified to near electrophoretic homogeneity by metal ion affinity chromatography. The antibacterial activity of the LysC variants against *S. aureus* ATCC 25923 is shown in Table 2. All of them demonstrated anti-*S. aureus* activity. Three variants of the conserved HHT motif—H50A, H51A, and T52A—caused bacterial reduction of 3.48 ± 0.30, 4.31 ± 0.19, and 2.75 ± 0.11 log units that corresponded to as much as 68.0%, 84.2%, and 53.7% of initial LysC activity, respectively. Out of these variants, H50 is directly involved in zinc coordination (Figure 6C). Variants that contain substitution of two other zinc binding residues: H147A and C155A, showed bactericidal activity at the levels of 33.2% ± 0.6 and 49.8% ± 2.0, respectively. The variant with the Y76A substitution had antibacterial activity of 80.5% ± 3.1. The lowest reduction in *S. aureus* cell number (1.65 ± 0.06 log units) was observed for the T153A variant, which represented 32.2% of the wild-type LysC activity. The results were surprising since we expected that all of the LysC substitution variants would significantly or completely lose their bactericidal activity. In general, alanine substitution of residues closer to the *C*-terminal region had a more pronounced effect on LysC activity than replacement of middle and *N*-terminal residues. Still, the activity of all substitution variants was quite high, bearing in mind the fact that substitutions of His50, His147, and Cys155 may abrogate zinc binding by the protein. Therefore, we were wondering what other features of the LysC protein may be responsible for its antibacterial activity.

### 2.6. In Silico Analysis of the LysC Protein in Search for Motifs Underpinning Antibacterial Activity

**Figure 7:**
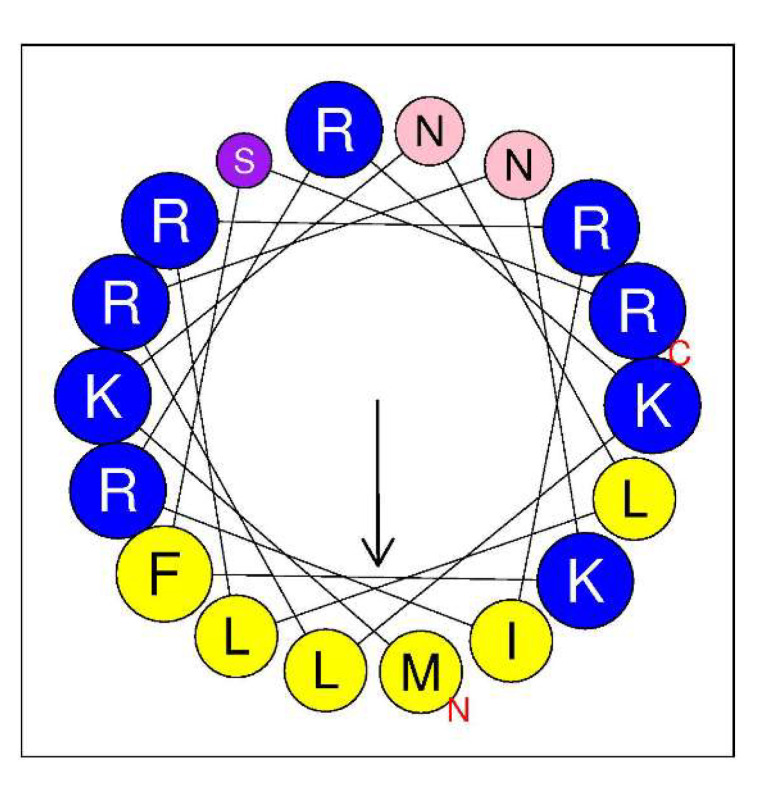
Helical wheel diagram of the *N*-terminal region of LysC. The projection was obtained from HeliQuest (http://heliquest.ipmc.cnrs.fr/cgi-bin/ComputParams.py). Predicted hydrophobic face includes I, M, L, L, and F. Positively charged residues are highlighted by blue color, while hydrophobic residues are marked in yellow. Polar uncharged residues are represented by purple (S) and pink (N). Size of the analysis window was 18 amino acids. The arrow represents the helical hydrophobic momentum.

AMPA, a web application for assessing the potential presence of antimicrobial domains in proteins, was used to identify any putative antimicrobial pattern in LysC [21]. Scanning LysC sequence with AMPA predicted, with 1% of misclassification probability, an antimicrobial region spanning from amino acids 2–19 of the protein. Therefore, we focused on the evaluation of the antibacterial potential of the *N*-terminal region of the enzyme. We assumed that the antimicrobial activity of the *N*-terminal region of LysC may be associated with its high positive charge, which may be responsible for interactions with negatively charged phosphate head groups of bacterial membranes. These types of protein regions with cationic amino acids are often found to adopt a α-helical structure [22]. Indeed, in the structure of LysC, lysine and arginine residues are often flanked by hydrophobic amino acids like methionine, isoleucine, leucine, and phenylalanine, and may form an amphipathic α-helical structure with antibacterial properties, as shown by helical wheel projection (Figure 7). The in silico analysis using the Antimicrobial Peptide Calculator and Predictor (APD3) supports predictions of the α-helical structure formation by the protein fragment comprising both the arginine-rich sequence (residues 1–24) at the *N*-terminus of LysC and additional seven residues that include lysine, isoleucine, and two valines (residues 1–31). The helical pattern with a hydrophobic ratio of 33% and net charge of +9 (Table S2) predicted by APD3 suggests that there may be eight residues on the hydrophobic surface of the α-helix and that the putative peptide may interact with bacterial membranes and act as an antimicrobial peptide.

### 2.7. Antibacterial Activity of LysCΔ2–23 and a Synthetic Peptide Derived from the N-Terminal Part of the Enzyme

**Figure 8:**
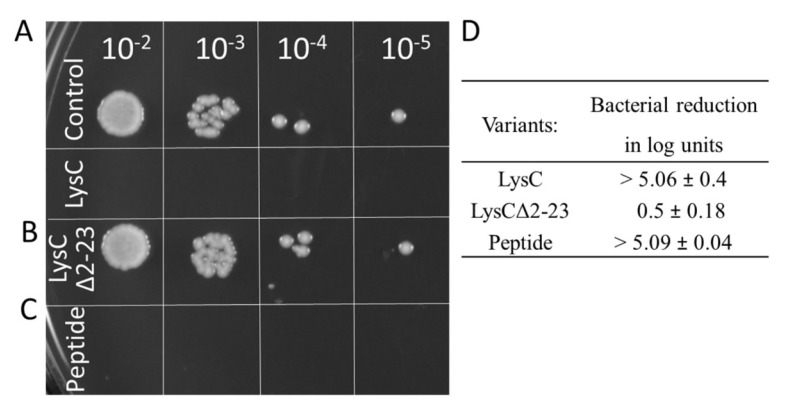
Antimicrobial activity of LysCΔ2–23 and the Intestinalin peptide against *S. aureus* ATCC 25923. Antibacterial test with LysC served as a positive control. Drops in a volume of 5 μL, containing serial 10-fold dilutions of (**A**) control samples and samples with LysC, (**B**) LysCΔ2–23 and (**C**) 30 aa peptides (Intestinalin), respectively, were spotted onto TSB agar. Plates were incubated overnight, and then photographed; **(D)** Antibacterial activities are expressed as bacterial reduction in logarithmic units relative to the untreated control (average from three replicate measurements ± standard deviation).

First, we removed the *N*-terminal extension of LysC (residues 2–23) with 10/23 positively charged residues (43.5%), creating a deletion variant of the enzyme, termed LysCΔ2–23. The zymogram assay showed that the LysCΔ2–23 retained less than 10% of wild-type protein activity as judged by densitometric analysis (data not shown). Next, as shown by the spot dilution assay, the addition of purified LysCΔ2–23 to bacterial cell suspensions, in comparison to the control to which LysC was added, did not cause any noticeable reduction in the number of viable cells (Figure 8A,B). The addition of 500 μg/mL LysCΔ2–23 reduced the viable cell number of *S. aureus* ATCC 25923 by only 0.5 ± 0.18 log units that stand for over a 10-fold decrease of antibacterial activity of the truncated variant (Figure 8D). Subsequently, based on the LysC amino acid sequence and results of analysis of the APD3 software, we synthesized a 30 amino acid peptide (amino acids 2–31, with 11 positively charged and 10 hydrophobic residues) derived from the *N*-terminal region of LysC to study its antibacterial potential. The peptide, which we termed Intestinalin, caused pronounced reduction of 5.09 ± 0.04 log units of viable *S. aureus* cell counts in the CFU reduction assay, a level comparable to the activity of wild-type LysC (Figure 8C,D). In addition, a much lower concentration of the peptide (20 μg/mL; 5 μM) was required to achieve bactericidal activity similar to that achieved by 500 μg/mL (21.4 μM) of LysC. The IC_50_ value for the peptide was determined to be 6 μg/mL (1.5 μM). These experiments clearly confirm that the *N*-terminal extension of LysC is involved in the antibacterial activity. However, bearing in mind the in silico results (Figure 7 and Table S2) we also wanted to verify the conformation of Intestinalin in the presence of bacterial membrane substitutes.

### 2.8. Secondary Structure of Intestinalin

**Figure 9:**
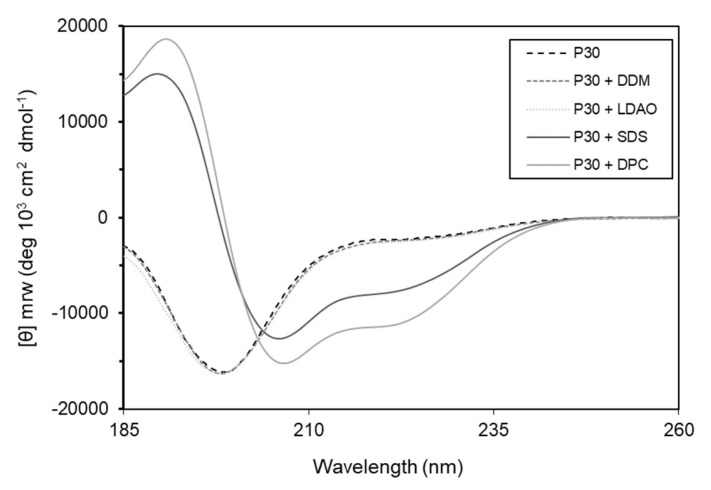
Circular dichroism spectra of P30 peptide show α-helical conformation of Intestinalin (P30) in the presence of detergents SDS and DPC (solid lines). Without addition of detergents or in the presence of DDM or LDAO (dotted lines) Intestinalin is in a randomly coiled state; DDM: lauryl-*β*-d-maltoside; DPC: *n*-dodecylphosphocholine; SDS: sodium dodecyl sulfate; LDAO: lauryldimethylamine *N*-oxide.

Many α-helical antibacterial peptides are unstructured in solution, but in the presence of membranes they adopt amphipathic helical structures [23]. To test this hypothesis, we used circular dichroism spectroscopy and investigated the secondary structure of the peptide without and in the presence of detergents that mimic bacterial membranes. The results show that the peptide, alone or in the presence of lauryl-*β*-d-maltoside (DDM) and lauryldimethylamine *N*-oxide (LDAO) is unstructured in solution. However, in the presence of sodium dodecyl sulfate (SDS) and *n*-dodecylphosphocholine (DPC) the peptide adopts α-helical conformation (Figure 9).

## 3. Discussion

The application of partially purified phage-associated endolysin to exogenously lyse the cell wall of groups A and C of hemolytic streptococci was first reported in 1958 [24]. Since then, many endolysins have been characterized and applied as antibacterial agents as an alternative to conventional antibiotics [25]. However, the success of antibacterial application of endolysins and antibacterial agents would not be possible without a comprehensive understanding of their biochemical, biophysical, and antibacterial properties [19,26,27]. The two endolysins under study in our laboratory, Ts2631 and Ph2119, which originate from *T. scotoductus* bacteriophages, reveal structural features characteristic for type 2 amidases (family: PF01510), with a Zn^2+^ ion coordinated by two histidines and a cysteine residue. Both these proteins show amino acid sequence and structure similarity to numerous enzymes from mesophilic bacteria, including the LysC lytic enzyme of *C. intestinale* URNW (Figure 1). LysC, as indicated by in silico analysis of its encoding gene and flanking DNA fragment, is not a phage protein, but probably represents an example of bacterial autolysins of the family of type 2 amidases (http://pfam.xfam.org/family/PF01510). It is less likely that LysC functions as a bacteriocin, because, as determined by SignalP software [28], the enzyme lacks a recognizable signal peptide that may facilitate its secretion. The other possibility is that the *lysC* gene is reminiscent of temperate phage infection, where most of the prophage sequences were lost over time and the protein adopted the role of a bacterial autolysin.

Well characterized examples of autolysins with type 2 amidase activity include AtlE from *S. epidermidis* with an amidase domain AmiE [29,30], and AmpD from *Citrobacter freundii* [17]. Both, AmiE (PDB entry: 3LAT) and AmpD (PDB entry: 2Y28) adopt a globular fold, with several α-helices surrounding the central β-sheet that makes them similar to LysC. These enzymes are also zinc-dependent amidases, coordinating a zinc atom by a His/His/Asp triad, although, in the amino acid sequence of LysC a cysteine residue replaces the aspartate [17,30]. However, even though these three proteins share the same fold, they also exhibit significant differences. AmiE is a part of the main *S. epidermidis* Autolysin E, AtlE [29], which is a modular protein comprised of a signal peptide, a pro-peptide, an amidase domain (AmiE), and a glucosaminidase domain linked to AmiE by three repeats (R1–R3) [29]. The second amidase, AmpD, is an enzyme which takes part in the cell-wall recycling pathway and as such has a strict specificity for 1,6-anhydro-muropeptides and does not recognize intact peptidoglycan [31]. Similarly to research on AmpD, we aim to reveal the substrate specificity of LysC and the regulatory mechanism of its potential amidase activity either by performing in silico docking studies or peptidoglycan digestion assays [30]. It is also possible, that having the pI of 9.96 and a highly positively charged *N*-terminal region, LysC destabilizes bacterial membranes by electrostatic interactions. This scenario was postulated for recently discovered TSPphg lysin [32].

The main objective of the present study was to characterize the newly discovered lytic enzyme, similar to Ts2631 endolysin in the context of its putative antimicrobial potential [14]. This goal was achieved by successful application of LysC against the high priority pathogen *S. aureus* (Figure 3 and Figure 4). So far, only few endolysin-like amidases have been tested as antibacterial agents. The best known example is the major pneumococcal autolysin LytA, which has been found to be active against a β-lactam-resistant *S. pneumoniae* in mice peritonitis-sepsis models [33]. LysC is active against *Clostridium* species, specifically *C. intestinale* and *C. sporogenes*, as well as *B. cereus, M. luteus,* and *S. aureus* (Figure 2). We revealed its anti-staphylococcal potential, which shows on average the 5.12 ± 0.14 bacterial log reduction after 1.5 h incubation with *S. aureus* cells at 37 °C (Figure 3C). However, the antibacterial application of other autolysins against *S. aureus* was moderately successful [34]. For example, LytM autolysin that cleaves pentaglycine crossbridges of *S. aureus* peptidoglycan was not efficient in combating *S. aureus* in mice models of chronic *S. aureus* infected eczema [34]. In addition, recombinantly produced *S. aureus* autolysin LytN showed low lytic activity when applied exogenously [35]. A more successful strategy included the use of molecular engineering techniques to fuse the cell wall binding domains (CBDs) to existing autolysins to increase their affinity to bacterial cell walls, thus enhancing their lytic activity [36]. Similar molecular engineering techniques were also applied in case of phage endolysins in order to improve their antibacterial potential [13,25,37,38]. For example, exchanging the CBD of Ply187 endolysin from *S. aureus* phage 187 with the CBD from staphylococcal phage K endolysin, LysK, improved bacteriolytic activity of the chimeric enzyme 10-fold over the parental enzyme, particularly against methicillin-resistant *S. aureus* strains [39]. Not only CBDs, but also Artilysins, were exchanged between lytic enzymes to enhance their antibacterial activity. Briers and colleagues coined the term Artilysins to describe a group of endolysin-based enzymes designed to penetrate the outer membrane of Gram-negative bacteria and to strengthen the interactions of lytic enzymes with the polyanionic cell surface of Gram-positive bacteria [12,13]. Several Artilysins were constructed by fusing endolysins with different outer membrane-destabilizing peptides such as polycationic nonapeptide (PCNP), hydrophobic pentapeptide (HPP) or α-helical cathelicidin (SMAP-29) [12,40]. The most efficient in increasing the antibacterial activity was the fusion of the *N*-terminus of endolysins with designed polycationic peptide (PCNP) [12].

In the present study we showed that LysC antibacterial activity is mediated by its highly cationic *N*-terminal region, as the LysC truncated variant, LysCΔ2–23, was inactive against *S. aureus* ATCC 25923 (Figure 8). Moreover, a synthetic peptide derived from the *N*-terminal region of LysC (Intestinalin) showed anti-staphylococcal activity at the level comparable to the wild-type enzyme (Figure 8). We also checked the antibacterial activity of Intestinalin using four models of the antimicrobial peptide prediction tool, known as the Collection of Anti-Microbial Peptides (CAMP). As seen in Table S3, all four tested models of the prediction tool classified the *N*-terminal region of LysC as an antimicrobial peptide (AMP) with high probability.

We show that in the presence of detergents that mimic bacterial membranes, Intestinalin adopts in silico analysis, α-helical structure, as predicted by HeliQuest, that is characteristic of polycationic antimicrobial peptides [23]. Like most of the α-helical peptides, LysC remains unstructured in aqueous solution [23,41], which is consistent with the circular dichroism spectroscopy data presented in Figure 9. Interestingly, the results of our crystallographic studies also show that the *N*-terminal region of LysC is an intrinsically disordered protein region (IDPR) (Figure 6). It is known that many IDPRs bind to artificial and natural membranes and this interaction has been shown to be accompanied by a dramatic increase in their α-helical content [42]. This is also in agreement with our results. We strongly believe that LysC may serve as a natural source of a polycationic amino acid sequence with a strong antibacterial activity. Recently, a novel DNA assembly method (VersaTile platform) was developed to create a practically unlimited number of shuffled lytic enzymes variants [38]. Fusion of the Intestinalin to other known or yet-to-be discovered lytic enzymes will open up new possibilities to increase their antibacterial potential and broaden the spectrum of their application.

## 4. Materials and Methods

### 4.1. Bacterial Strains, Plasmids, and Culture Conditions

*Escherichia coli* DH5α and *E. coli* BL21(DE3) strains were used for cloning and recombinant proteins production, respectively. The pET15b expression plasmid used for cloning of the *lysC* gene was purchased from Merck, Darmstadt, Germany. *E. coli*, *B. cereus* ATCC 13061, *B. subtilis* 168 DSM 23778, and *M. luteus* ATCC 4698 were grown aerobically in Luria–Bertani (LB) broth at 37 °C. *S. aureus* ATCC 25923 and *S. aureus* ATCC 6538P were grown in tryptic soy broth (TSB) (Graso Biotech, Starogard Gdanski, Poland). When necessary, LB was supplemented with 100 μg/mL of ampicillin. *C. intestinale* DSM 6191 and *C. sporogenes* DSM 767 were grown anaerobically in TSB at 37 °C without shaking.

### 4.2. DNA Manipulations

Standard protocols for molecular cloning, polymerase chain reaction (PCR), DNA analysis, and bacterial transformation were applied [43]. The *lysC* gene coding for LysC lytic enzyme was synthesized by GeneArt Gene Synthesis Service (Life Technologies, Regensburg, Germany) and amplified by PCR with use of primers listed in Table S1, specifically lysC-nde-f and lysC-bam-h-r. The PCR product was digested with NdeI and BamHI enzymes and cloned into the corresponding sites of pET-15b vector to generate pET-LysC. Primer LysCΔ2–23 instead of lysC-nde-f was used to generate by PCR and to clone into pET15b, a truncated version of *lysC* gene yielding plasmid pET-LysCΔ2–23. This resulted in the introduction of a His-tag moiety to *N*-terminus of each recombinant enzyme. Mutations in seven codons of *lysC* gene were independently introduced by PCR according to the instruction manual of the QuikChange II Site-Directed Mutagenesis kit (Agilent Technologies, Santa Clara, CA, USA). The mutagenic oligonucleotides are shown in Table S1. PCRs for single codon mutations were run with the use of pET-LysC as a template. The resulting mutant variants were verified by DNA sequencing. Plasmids constructed in this study were deposited in the Collection of Plasmids and Microorganisms, KPD, University of Gdansk, Gdansk, Poland.

### 4.3. Overproduction and Purification of Recombinant Proteins

The overnight cultures of *E. coli* BL21(DE3) strain harboring the recombinant plasmids (pET-LysC and pET-LysCΔ2–23) constructed as described above or pET-15b vector harboring *ph2119* gene, termed pMP20 [9], were diluted 100-fold in 1 L of fresh LB broth and grown to OD_600_ = 0.5. Then, protein overproduction was induced with isopropyl-β-d-thiogalactopyranoside (IPTG) brought to 1 mM and carried out for 4 h at 37 °C. Cells were harvested, suspended in 30 mL of NPI-10 buffer (50 mM NaH_2_PO_4_, pH 8.0, 300 mM NaCl, 10 mM imidazole, 0.1% Triton X-100, 10% (*v/v*) glycerol) and disrupted by sonication on ice (30 bursts of 10 s at an amplitude of 12 μm). The cell lysates were centrifuged at 11,000× *g* for 20 min at 4 °C. Supernatants with His-tagged recombinant proteins were applied to a cobalt-based immobilized Metal Affinity Chromatography (IMAC) with 1 mL HiTrap TALON crude columns (GE Healthcare), washed with NPI buffer containing 20 mM imidazole and eluted with the NPI buffer without 0.1% Triton X-100, containing 150 mM imidazole. LysC purity was further analyzed by size exclusion chromatography (SEC) on a Superdex 75 10/300 GL column (GE Healthcare, Chicago, IL, USA) equilibrated with buffer A (50 mM NaH_2_PO_4_, 300 mM NaCl, pH 8.0). Sodium dodecyl sulfate-polyacrylamide gel electrophoresis (SDS-PAGE) was used to assess protein homogeneity. Fractions containing pure proteins were dialyzed against 20 mM MES, pH 6.0 for crystallography studies or against 20 mM HEPES, pH 7.4 for activity testing. Protein concentration was determined using the Bradford assay [44].

### 4.4. Bioinformatics Analysis

Protein sequences were aligned with CLUSTAL Omega (software available at http://www.clustal.org/omega/) [45]. PHASTER was utilized for the identification of prophage sequences within the genome of *C. intestinale* URNW [18]. The AMPA web software (http://tcoffee.crg.cat/apps/ampa) was applied in a search for LysC regions with antibacterial potential. The antibacterial properties of the *N*-terminal extension of LysC and physicochemical properties of the 30 aa peptide (Intestinalin), including amino acid composition, molecular weight, hydrophobic ratio, net charge, and protein-binding potential were predicted with the use of Antimicrobial Peptide Calculator and Predictor (APD3) (http://aps.unmc.edu/AP/prediction/prediction_main.php) [46]. Helical wheel projection of the 18 amino acid *N*-terminal regions of LysC was generated using HeliQuest [47]. The antimicrobial activity of the synthetic peptide Intestinalin was predicted by the Collection of Anti-Microbial Peptides (CAMP) (available at http://www.camp.bicnirrh.res.in/predict/) [48]. This tool is based on four different algorithms: support vector machine (SVM), random forest (RF), artificial neural network (ANN), and discriminant analysis (DA). As a result, three of these models, namely, SVM, RF, and DA gave a probability score between 0 and 1, where values above 0.5 mean that the peptide is antimicrobial (AMP) and such information is automatically shown in the results window. The ANN model predicts peptides to be antimicrobial or not antimicrobial (AMP or NAMP).

### 4.5. Crystallization, Data Collection, and Structure Determination

Ph2119 endolysin crystals were grown at 4 °C from a solution of 8 mg/mL Ph2119 in 20 mM MES, pH 6.0. Crystals for data collection were obtained using seeding from crystals grown at similar conditions (20 mM NaKHPO_4_ and 16% (*w*/*v*) polyethylene glycol 3350) in sitting drops in MRC 3-well plates with 200 nL of protein, 50 nL seed, and 150 nL reservoir solution (30 mM NaKHPO_4_ and 16% (*w*/*v*) PEG 3350). LysC crystals were grown in a similar way at 20 °C from 200 nL 8.8 mg/mL LysC protein in 10 mM HEPES, pH 7.4 mixed with 200 nL reservoir (10 mM NaKHPO_4_ and 22% (*w*/*v*) PEG 3350). For data collection the crystals were transferred to cryo-solutions containing the reservoir and protein buffers solutions supplemented with 25% (*v*/*v*) glycerol, and flash-frozen in liquid nitrogen. Data to 1.2 Å were collected at Diamond Light Source beamLine i04. The data were processed using the automatic script xia2-3dii [49] with the XDS package [50] and AimLess [51]. The structure of Ph2119 endolysin was determined using automated molecular replacement method with the MrBUMP software package [52]. The peptidoglycan recognition protein from *Drosophila melanogaster* with PDB entry: 2F2L was found to be a successful template structure. The Ph2119 structure was refined using Refmac5 [53] for convergence and model building was done using Coot graphics software [54]. The final model includes amino acids 16–169, one Zn^2+^ atom, one phosphate group, and one glycerol molecule. The structure of LysC was determined using molecular replacement with MrBUMP software and Ph2119 as a template. The structure was refined and built similarly to Ph2119, and the final model contained amino acids 44–192, one Zn^2+^ atom, two phosphate groups, and two glycerol molecules.

### 4.6. Protein Sequences Accession Numbers

The structures were deposited with the Protein Data Bank with PDB codes: 6SU5 and 6SSC for the Ph2119 and LysC structures, respectively. The GenBank accession numbers for the protein sequences of the LysC lytic enzyme, Ph2119 endolysin, Ts2631 endolysin, PhiKo lysozyme, *Clostridium perfringens N*-acetylmuramoyl-l-alanine amidase, *Mus musculus* Pglyrp2, and *Drosophila hydei* PGRP-SC1a/b-like are ERK30183.1, AHF20915.1, AIM47292.1, AYJ74695.1, PWX13837.1, AAH19396.1, and XP_023167292.1, respectively.

### 4.7. Zymogram Assay

Zymogram analysis was performed by using 12.5% SDS-PAGE containing 0.2% (*w*/*v*) Gram-positive bacterial substrates in a separating gel. After electrophoresis, the gel was gently shaken at 37 °C for 16 h in 50 mL of 25 mM potassium phosphate buffer, pH 8.0 containing 1% Triton X-100 to allow renaturation of LysC. A clear band resulting from lytic activity was visualized after staining with 1% (*w*/*v*) methylene blue in 0.01% (*w*/*v*) KOH and subsequent destining with distilled water. Bovine serum albumin (Sigma-Aldrich, St. Louis, MO, USA) served as a negative control. The 12.5% SDS-PAGE without bacterial cells was run simultaneously and stained with Coomassie Brilliant Blue (Bio-Rad, Hercules, CA, USA).

### 4.8. Peptide Synthesis

The peptide, which we named Intestinalin, was synthesized on a solid phase using a Liberty Blue microwave synthesizer (CEM Inc.). Tenta Gel R RAM resin of 0.19 mmol/g substitution (Rapp Polymere) was used as a solid support. The synthesis procedure consisted of repeatedly alternating deprotection and coupling steps, according to the manufacturer’s protocols (Liberty Blue^TM^ User Guide). Deprotection was executed with 20% piperidine. Subsequent acylation of the free amine group with a protected amino acid residue was carried out using diisopropylcarbodiimide/Oxyma activation method. A mixture consisting of 88% trifluoroacetic acid, 2% triisopropylsilane, 5% water and 5% thioanisole was used for the cleavage of the peptide from the resin and side chains deprotection. The product was precipitated with cold diethyl ether and washed three times with the same solvent. Next, the peptide was dissolved in water and lyophilized. The identity of the peptide was confirmed by mass spectra recorded with a Biflex MALDI TOF spectrometer (Bruker). Purification of the peptide was carried out by a semi-preparative reverse phase high-performance liquid chromatography (RP-HPLC) using a Jupiter Proteo C12 column (21.2 × 250 mm, 4 μm, 90 Å; Phenomenex). Chromatographic analyses of the crude and pure peptide were performed on an RP-HPLC Varian Pro Star 240 using a Kromasil C8 column (4.6 × 250 nm, 5 μm, 100 Å).

### 4.9. Antimicrobial Activity in Vitro

*S. aureus* ATCC 25923 and *S. aureus* ATCC 6538P cells were grown in TSB at 37 °C to exponential phase (OD_600_ ~0.45–0.5). Bacteria were harvested at 4000× *g* for 15 min at 20 °C, washed with 20 mM HEPES, pH 7.4 and resuspended in the same buffer. For the antimicrobial activity assay, LysCΔ2–23, LysC or its substitution variants were added at a final concentration of 500 μg/mL to approximately 10^6^ of bacterial cells (10-fold dilution) in 20 mM HEPES, pH 7.4 in a final volume of 300 μL. In case of Intestinalin the final concentration of the peptide was 20 μg/mL. To the negative control, the equivalent volume of 20 mM HEPES, pH 7.4 was added instead of LysC enzyme or its derivatives. After 1.5 h of incubation at 37 °C, reaction mixtures were serially diluted in 20 mM HEPES, pH 7.4 and plated. Simultaneously, 5 μL drops were plated onto TSB agar plates to perform spot dilution assays. Colony forming units (CFUs) were counted after overnight incubation at 37 °C. The antibacterial activity was presented in logarithmic units = log_10_ (N0/Ni), where *N0* = the number of untreated cells (in the negative control) and *Ni* = number of treated cells counted after incubation. All experiments were performed in triplicate.

### 4.10. Transmission Electron Microscopy

LysC at a final concentration of 500 μg/mL was mixed with 10^8^ of *S. aureus* ATCC 25923 cells in a volume of 500 μL of 20 mM HEPES, pH 7.4. In the control reaction, equivalent volume of 20 mM HEPES, pH 7.4 was added to the bacterial cell suspension instead of the enzyme. Samples were incubated for 1.5 h at 37 °C, washed in phosphate buffer saline (PBS) (Sigma-Aldrich) and fixed overnight at 4 °C in 2.5% glutaraldehyde in PBS. Following rinsing, samples were post-fixed in 1% OsO_4_ for 1 h at room temperature. After dehydration in a graded series of ethanol, probes were embedded in EPON resin, cut on an ultramicrotome Leica UC7, counterstained with uranyl acetate and lead citrate, and viewed with a FEI Tecnai BioTwin Spirit microscope (FEI Company, Hillsboro, Oregon, United States).

### 4.11. Circular Dichroism Spectroscopy

The secondary structure of Intestinalin was studied by circular dichroism (CD) spectroscopy. A J-815 spectropolarimeter (Jasco) with a 1-mm path length cell was used to record the spectra of Intestinalin (40 μM) at 25 °C from 185 to 260 nm at 0.1 nm intervals. The peptide was dissolved in water or 20 mM micelles of lauryl-*β*-d-maltoside (DDM), dodecylphosphocholine (DPC), lauryldimethylamine *N*-oxide (LDAO), and sodium dodecyl sulfate (SDS). Each spectrum represents the average of six scans. CD data are shown as the mean residue ellipticity (θ) in degrees·cm^2^·dmol^−1^.

## Figures and Tables

**Table 1:** Data collection and refinement statistics.

Data Collection		Ph2119 PDB ID: 6SU5	LysC PDB ID: 6SSC
Space group		P2_1_2_1_2_1_	P2_1_2_1_2_1_
Cell Dimensions	a, b, c (Å)	32.0, 62.6, 74.5	37.4, 53.8, 76.7
	α, β, γ (°)	90, 90, 90	90, 90, 90
Wavelength (Å)		0.97954	0.97951
Resolution (Å)		62.6–1.17 (1.19–1.17) *	14.90–1.21 (1.23–1.21) *
R_merge_		0.070 (0.65)	0.066 (1.11)
Mean I/σ(I)		8.1 (1.4)	12.1 (1.3)
Completeness (%)		99.1 (94.9)	99.8 (99.8)
Redundancy		4.3 (3.7)	5.2 (5.2)
Refinement (Å)		48–1.20	14.9–1.21
R_work_/R_free_		0.144/0.189	0.121/0.161
No. of atoms		1490	1467
Ligands		1 Zn^2+^, 1 PO_4_, 1 glycerol	1 Zn^2+^, 1 PO_4_, 2 glycerol
No. of water		232	231
RMSD of bonds (Å)		0.016	0.018
RMSD of angles (°)		2.0	2.2
Ramachandran	Favored regions (%)	96.9	97.5
	Allowed regions (%)	2.5	2.5
	Outliers (%)	0.6	0.0

* Numbers in brackets refer to the highest resolution shells. RMSD: root-mean-square deviation; PDB: Protein Data Bank.

**Table 2:** Antimicrobial activity of LysC variants. Activities are expressed as bacterial reduction in logarithmic units and as percentage in relation to the native LysC control (average from three replicate measurements ± standard deviation).

Variants	Bacterial Reduction in log Units	Relative Activity (%)
LysC	5.12 ± 0.14	100 ± 2.7
H50A	3.48 ± 0.30	68.0 ± 5.9
H51A	4.31 ± 0.19	84.2 ± 3.7
T52A	2.75 ± 0.11	53.7 ± 2.1
Y76A	4.12 ± 0.16	80.5 ± 3.1
H147A	1.70 ± 0.03	33.2 ± 0.6
T153A	1.65 ± 0.06	32.2 ± 1.2
C155A	2.55 ± 0.10	49.8 ± 2.0

## Supplementary Materials

Figure S1. Size-exclusion chromatography analysis of LysC, Table S1. PCR primers used in this study, Table S2. Physicochemical properties of *N*-terminal region of LysC and Table S3. Antimicrobial peptide prediction of the N-terminal region of LysC. Supplementary materials can be found at https://www.mdpi.com/1422-0067/21/14/4894/s1.
