# Supplementary material for: Molecular Characterization of a Novel Lytic Enzyme LysC from Clostridium intestinale URNW and Its Antibacterial Activity Mediated by Positively Charged N-Terminal Extension
Source: Int J Mol Sci. 2020 Jul 11;21(14):4894. doi: 10.3390/ijms21144894 (PMC7404271; doi:10.3390/ijms21144894)
Supplement: Supplementary file 1 [file ijms-21-04894-s001.pdf]

Supplementary Materials

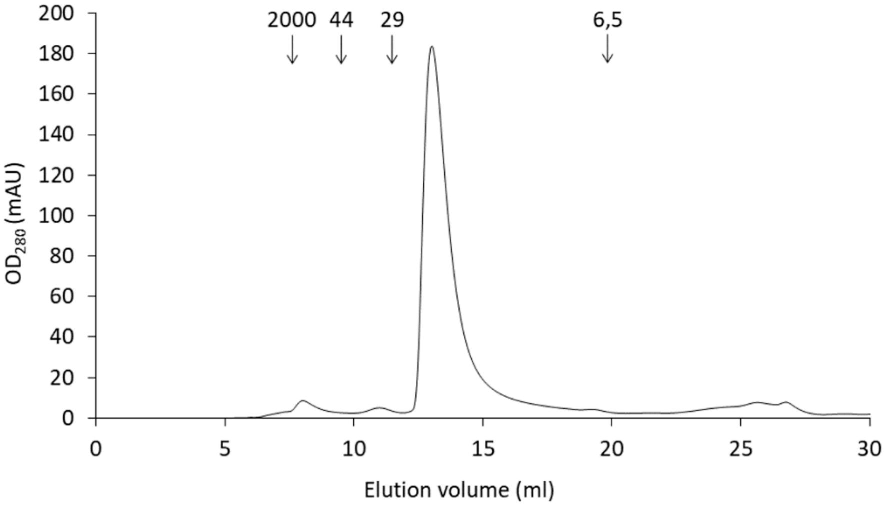

Figure S1. Size-exclusion chromatography analysis of LysC. Chromatography was carried out on a Superdex 75 10/300 GL column equilibrated with 50 mM NaH_2PO_4, 300 mM NaCl, pH 8.0 at a flow rate of 0.8 mL/min. LysC was eluted in a single peak at 13.02 mL using UV detection at 280 nm (mAU, milli-absorbance units). The positions of molecular weight standards are marked as arrows: Blue Dextran 2000 (M_r = 2,000,000); Ovalbumin (M_r = 44,000); Carbonic Anhydrase (M_r = 29,000); Ribonuclease A (M_r = 6,500).

Table S1. PCR primers used in this study.

Primer Name	Oligonucleotide Sequences (5´→ 3´)	Annealing Temperature
	**Cloning of *lysC* gene**	
lysC-nde-f	ACGACCATATGAAAAATCTGCTGCG	50 °C
lysC-bam-h-r	GAGCGGATCCTTATTTCACGTTCA	50 °C
lysCΔ2-23	ACGACCATATGCCGAAAATTGTGGA	56 °C
	**Site-directed mutagenesis of *lysC* gene**	Codon change:
H50A-F	CTATAACTTTAATCCGAACATGATCGTGTATGCTCACACCGTGGATAAT	CAT → GCT
H50A-R	ATTATCCACGGTGTGAGCATACACGATCATGTTCGGATTAAAGTTATAG	CAT → GCT
H51A-F	AACTTTAATCCGAACATGATCGTGTATCATGCCACCGTGGATAATAAC	CAC → GCC
H51A-R	GTTATTATCCACGGTGGCATGATACACGATCATGTTCGGATTAAAGTT	CAC → GCC
T52A-F	CATGATCGTGTATCATCACGCCGTGGATAATAACATGAC	ACC→ GCC
T52A-R	GTCATGTTATTATCCACGGCGTGATGATACACGATCATG	ACC→ GCC
Y76A-F	CAGCGTGGTTGGAGCGGTATTGGTGCTCATTTCTATATTCGTAAA	TAT → GCT
Y76A-R	TTTACGAATATAGAAATGAGCACCAATACCGCTCCAACCACGCTG	TAT → GCT
H147A-F	TATCACCGATCTGAAACGCGCTAAAGATGTTCGTCAGACC	CAT → GCT
H147A-R	GGTCTGACGAACATCTTTAGCGCGTTTCAGATCGGTGATA	CAT → GCT
T153A-F	CATAAAGATGTTCGTCAGGCCGAATGTCCGGGTAATA	ACC → GCC
T153A-R	TATTACCCGGACATTCGGCCTGACGAACATCTTTATG	ACC → GCC
C155A-F	CCATAAAGATGTTCGTCAGACCGAAGCTCCGGGTAATAACT	TGT → GCT
C155A-R	AGTTATTACCCGGAGCTTCGGTCTGACGAACATCTTTATGG	TGT → GCT

Primers for cloning of lysC gene were design with use of Primer3plus software. Primers for site-directed mutagenesis of lysC gene were design with use of QuikChange Primer Design Program (http://www.genomics.agilent.com). Underlined sequences indicate cleavage sites for restriction enzymes NdeI and BamHI, respectively.

Table S2. Physicochemical properties of *N*-terminal region of LysC.

	**Intestinalin**
Amino acid	30
Molecular weight	3678
Amino acid composition	Hydrophobic amino acid - I: 3, V: 3, L: 3, F: 1, C: 0, M: 0, A: 0, W: 0<br>The number of G and P - G: 0, P: 1,<br>Negatively charged amino acid - E: 1, D: 1,<br>Positively charged amino acid - K: 5, R: 6, H: 0<br>Other amino acid - T: 1, S: 2, Y: 0, Q: 0, N: 3
Hydrophobic ratio	33%
Net charge	+9
Protein-binding potential (Boman index)	3.91 kcal/mol

Table S3. Antimicrobial peptide prediction of the *N*-terminal region of LysC.

**Peptide Sequence**	**KNLLRRIRRKLRNKFSRSDVIKTPKIVEVN**	
	**Class**	**AMP probability**
Support Vector Machine (SVM) classifier	AMP	0.979
Random Forest Classifier	AMP	0.733
Artificial Neural Network (ANN) classifier	AMP	
Discriminant Analysis classifier	AMP	0.995

CAMP is available at http://www.bicnirrh.res.in/antimicrobial; AMP—antimicrobial peptide.

© 2020 by the authors. Licensee MDPI, Basel, Switzerland. This article is an open access article distributed under the terms and conditions of the Creative Commons Attribution (CC BY) license (http://creativecommons.org/licenses/by/4.0/).
